# Health knowledge and self-efficacy to make health behaviour changes: a survey of older adults living in Ontario social housing

**DOI:** 10.1186/s12877-022-03116-1

**Published:** 2022-06-01

**Authors:** Jasmine Dzerounian, Melissa Pirrie, Leena AlShenaiber, Ricardo Angeles, Francine Marzanek, Gina Agarwal

**Affiliations:** 1 Department of Family Medicine, McMaster University, 100 Main St W, Hamilton, ON L8P 1H6 Canada; 2 Department of Health Research Methods, Evidence, and Impact, McMaster University, 1280 Main St W, Ontario L8S 4K1 Hamilton, Canada

**Keywords:** Older adults, Vulnerable, Social housing, Chronic disease, Health knowledge, Self efficacy, Health behaviour, Primary care

## Abstract

**Background:**

Older adults living in social housing are a vulnerable population facing unique challenges with health literacy and chronic disease self-management. We investigated this population’s knowledge of cardiovascular disease and diabetes mellitus, and self-efficacy to make health behaviour changes (for example, physical activity). This study characterized the relationship between knowledge of health risk factors and self-efficacy to improve health behaviours, in order to determine the potential for future interventions to improve these traits.

**Methods:**

A cross-sectional study (health behaviour survey) with adults ages 55+ (*n* = 599) from 16 social housing buildings across five Ontario communities. Descriptive analyses conducted for demographics, cardiovascular disease and diabetes knowledge, and self-efficacy. Subgroup analyses for high-risk groups were performed. Multivariate logistic regressions models were used to evaluate associations of self-efficacy outcomes with multiple factors.

**Results:**

Majority were female (75.6%), white (89.4%), and completed high school or less (68.7%). Some chronic disease subgroups had higher knowledge for those conditions. Significant (*p* < 0.05) associations were observed between self-efficacy to increase physical activity and knowledge, intent to change, and being currently active; self-efficacy to increase fruit/vegetable intake and younger age, knowledge, and intent to change; self-efficacy to reduce alcohol and older age; self-efficacy to reduce smoking and intent to change, ability to handle crises, lower average number of cigarettes smoked daily, and less frequent problems with usual activities; self-efficacy to reduce stress and ability to handle crises.

**Conclusions:**

Those with chronic diseases had greater knowledge about chronic disease. Those with greater ability to handle personal crises and intention to make change had greater self-efficacy to change health behaviours. Development of stress management skills may improve self-efficacy, and proactive health education may foster knowledge before chronic disease develops.

## Background

Despite advances in healthcare and chronic disease treatment and management, cardiovascular disease (CVD) [1] and type 2 diabetes mellitus (DM) [2, 3] prevalence rates have increased in the past few decades. Compared to younger populations, there is an increased prevalence of CVD and DM among older adults as well as mortality due to these conditions [1, 3, 4]. In Ontario alone, in 2015, over 28,000 deaths were attributed to CVD and DM combined; furthermore, deaths from DM were likely underestimated because DM is a risk factor for death due to other conditions [5]. In low-income older adults living in Ontario social housing, 96.7% have moderate-to-high risk of developing diabetes based on lifestyle-related modifiable risk factor assessments, and undiagnosed prediabetes and diabetes prevalence is likely close to 32.0% [6]. Moreover, the majority of older adults in Ontario social housing have either undiagnosed or unmanaged hypertension, and a high prevalence of modifiable risk factors for hypertension [7]. There is substantial opportunity to decrease the risk of poor outcomes due to CVD and DM in this population through chronic disease self-management, such as improving physical activity and eating behaviours and reducing smoking, alcohol consumption, and stress.

Development of self-management skills can reduce older adults’ risk of chronic disease development and associated complications. For example, in a study of American adults with diabetes, and an average of nearly five additional chronic illnesses, patients who practiced self-management through diet and medications reduced their CVD risk by 44 and 39%, respectively [8]. Three critical factors in chronic disease self-management are health literacy [9, 10], especially for older adult patients [11], health knowledge [12], and self-efficacy to make behavioural changes [12–14]. Previous research has found that 82% of older adults in social housing have inadequate health literacy [15], which is a major concern for chronic disease prevention and management. This also suggests that this population may have gaps in health knowledge, but the specific health knowledge gaps related to chronic disease remain unknown. Furthermore, no quantitative research studies have been identified that examine the level of self-efficacy to enact health behaviour changes in this population.

According to social cognitive theory, an individual’s degree of self-efficacy determines whether they will initiate a behaviour and how long they will sustain the behaviour in the face of barriers or adversity [16]. Two of the mechanisms that increase self-efficacy are previous mastery (personal experience) and social modelling (vicarious experience) [16, 17]. For older adults in social housing, 46% are insufficiently active, 34% eat less than one serving of fruit/vegetables per day, and 31% are current smokers [18], suggesting that many residents have limited personal experience with achieving desired health behaviours. Similarly, since these individuals are residing in close proximity, often clustered within apartment buildings, there may be limited social modelling of healthy behaviours. Housing has been established as a social determinant of health [19] and as a population, older adults living in social housing face the challenges of not only older age, but also experience challenges posed by low education, low health literacy, low income, and poor mobility, which can limit access to health education, primary care, and other health resources [15, 20]. Thus, engaging in beneficial health behaviours is not the social norm in this environment [18]. Therefore, older adults living in social housing are expected to have a low degree of self-efficacy to make health behaviour changes.

The aims of this exploratory study are to better understand the CVD and DM health knowledge of this population, the level of self-efficacy for health behaviour change, and the personal factors associated with self-efficacy for individuals residing in a social housing environment (i.e., age, gender, weight status, previous experience with health behaviours, health related knowledge, quality of life, health related stressors and ability to cope, intention to complete the behaviour). It is plausible that health knowledge may differ according to the individual’s current health behaviours and their disease status (e.g., an individual who has been diagnosed with diabetes may have had additional education from a healthcare provider). Also, chronic disease and other lifestyle risk factor status may influence self-efficacy for behaviour change (e.g., an individual who has been able to improve their physical activity may have a higher sense of mastery and thus increased self-efficacy for other behaviour changes [16]. Therefore, both health knowledge and self-efficacy will be examined in this exploratory study according to chronic disease and risk factor subgroups. Addressing this knowledge gap is important to inform future health interventions aiming to improve chronic disease self-management among low-income older adults in social housing by increasing their health knowledge and self-efficacy to make behaviour change.

## Methods

### Study design

This cross-sectional study aimed to describe knowledge of chronic disease risk factors and self-efficacy to make health behaviour change among older adults living in social housing in Ontario. This health behaviour data was gathered from a survey with adults (*n* = 599) ages 55+ from 16 social housing buildings across five Ontario communities (Hamilton, Guelph, Simcoe County, Sudbury, and York Region). This baseline participant data was collected in 2014/2015 as pre-intervention surveying prior to implementing a community program [21], the description of which is beyond the scope of this paper.

### Participant recruitment

In the 16 seniors’ social housing buildings, all building residents were invited to participate in the study through multiple modes of communication, including posters in common spaces, invitation letters in mailboxes, presentations at tenant meetings, invitation through tenant newsletter, and word of mouth. The study had two inclusion criteria: being at least 55 years old and residing in the building. Residents who could not communicate in English were excluded. Participants were provided a $10 grocery gift card as an honorarium. The Hamilton Integrated Research Ethics Board approved this study (Project #12-336), participation was voluntary, and all participants provided written consent.

### Data collection

Data were collected through face-to-face interviews by trained research staff and paramedics. To facilitate maximum participation among this population, surveying was held in a common space within the building on a drop-in basis (i.e., no need to reply to the invitation or book an appointment, familiar and accessible location that did not require travel, no need to have someone come into their home). Surveying was conducted on multiple days at each site in coordination with the housing provider (e.g., avoided grocery bus days). Consecutive sampling was used whereby surveying sessions continued to be held within each building until there were very few or no attendees. Five hundred ninety-nine older adults completed the surveys and were included in this study. A full description of the demographics, risk factors, and other participant factors of the sample are found in Tables 1, 2 and 3.

**Table 1:** Participant demographics

Demographic Characteristic	Response	Frequencies (%)	n
Gender	Female	453 (75.6)	599
	Male	146 (24.4)	
Age	55-64	152 (25.4)	599
	65-74	233 (38.9)	
	75-84	158 (26.4)	
	> 84	56 (9.3)	
	Mean (SD) = 72.24 (8.61)		
Ethnicity	White	487 (89.4)	545
	Non-White	58 (10.6)	
Education Level	High school diploma / some high school	409 (68.7)	595
	Any post-secondary education	186 (31.3)	
Marital Status	Married	46 (7.7)	595
	Separated / Divorced / Widowed	460 (77.3)	
	Never Married	89 (15.0)	
Lives Alone	Yes	540 (90.8)	595
	No	55 (9.2)	
Has a Family Doctor	Yes	553 (92.3)	599
	No	46 (7.7)	

The full sample was 599 participants and thus n’s below 599 are due to missing values

**Table 2:** Participant risk factors

Risk Factor	Response	Frequencies (%)	n
BMI Category	Obese	205 (36.6)	560
	Overweight	183 (32.7)	
	Normal	155 (27.7)	
	Underweight	17 (3.0)	
Drinking status	Current Drinker	140 (24.6)	570
	Current Non-drinker	430 (75.4)	
Smoking status	Current Smoker	183 (32.3)	567
	Current Non-smoker	384 (67.7)	
Physical Activity	Low physical activity (less than 30 min/day)	273 (46.1)	592
	Adequate physical activity (30 min/day or more)	319 (53.9)	
Fruit/Vegetable intake	Not every day	198 (33.3)	594
	Every day	396 (66.7)	
Chronic Disease and Associated Events			
Heart Disease	Yes	171 (28.5)	599
	No	428 (71.5)	
Hypertension	Yes	326 (54.4)	599
	No	273 (45.6)	
High Cholesterol	Yes	227 (37.9)	599
	No	372 (62.1)	
Stroke	Yes	77 (12.9)	599
	No	522 (87.1)	
Diabetes	Yes	165 (27.5)	599
	No	434 (72.5)	
Quality of Life Indicators			
Mobility problems	Yes	347 (60.8)	571
	No	224 (39.2)	
Self-care problems	Yes	113 (19.9)	569
	No	456 (80.1)	
Problems doing usual activities	Yes	245 (43.0)	570
	No	325 (57.0)	
Pain or discomfort issues	Yes	405 (70.9)	571
	No	166 (29.1)	
Anxiety or depression	Yes	262 (46.0)	570
	No	308 (54.0)	
Health-related Stressors			
Own physical health problem or condition	Yes	272 (45.4)	599
	No	327 (54.6)	
Own emotional or mental health problem or condition	Yes	170 (28.4)	599
	No	429 (71.6)	

The full sample was 599 participants and thus n’s below 599 are due to missing values

**Table 3:** Other participant factors

**Participant Factor**	**Response**	**Frequencies (%)**	**n**
Intention indicated:			
To begin/increase physical activity	Yes	286 (50.8)	563^a^
	No	277 (49.2)	
To increase fruits/vegetables intake	Yes	141 (25.1)	562^a^
	No	421 (74.9)	
To reduce alcohol consumption	Yes	17 (13.6)	125^b^
	No	108 (86.4)	
To quit smoking/reduce amount smoked	Yes	67 (38.7)	173^c^
	No	106 (61.3)	
To reduce stress	Yes	94 (16.7)	563^a^
	No	469 (83.3)	
**Other Predictors/Risk Factors**	**Mean (SD)**		**n**
Rating of ability to handle personal crises (5-point Likert scale)	3.13 (1.21)		597^a^
Rating of ability to handle day-to-day stress (5-point Likert scale)	3.43 (1.08)		598^a^
Number of cigarettes smoked in an average day	17.45 (11.70)		177^c^

^a^The full sample was 599 participants and thus n’s below 599 are due to missing values

^b^Restricted to those who indicated they are drinkers (*n* = 140) and thus n’s below 140 are due to missing values

^c^Restricted to those who indicated they are smokers (*n* = 183) and thus n’s below 183 are due to missing values

### Measures

All data was collected using the Health Awareness and Behaviour Tool (HABiT) [22]. The HABiT survey was developed as part of CP@clinic’s impact evaluation. It is reliable, has internal consistency, and content and face validity in the target population (older adults living in social housing) [22]. It assesses individuals’ health indicators such as health status and healthcare utilization and contains scales for measuring health knowledge, health behaviours and risk factors, health-related quality of life (HRQoL), risk perception and understanding, and self-efficacy to make health behaviour change [22]. Questions were developed and included from 15 sources, but primarily three surveys: the Canadian Community Health Survey [23], Canadian Diabetes Risk Questionnaire (CANRISK) [24], and a survey by the Canadian Hypertension Education Program [25]. In the current study sample, internal consistency measured using Cronbach’s alpha was 0.75 for knowledge and 0.60 for self-efficacy.

#### Current health behaviours and risk factors

Body Mass Index (BMI) was calculated based on self-reported height and weight and then categorized. Waist circumference was measured by research staff and/or research assistants conducting the interview. Where participants did not consent to waist circumference measurements, clothing/pant size was used as a proxy value. All other risk factors were self-reported by participants via the HABiT questionnaire [22]. All participants who self-reported any alcohol consumption or that they were an “Occasional” or “Daily” smoker were coded as ‘drinkers’ and ‘smokers,’ respectively. Numbers of cigarettes for smokers were self-reported. Likewise, participants self-reported their daily amount of physical activity, daily fruit/vegetable intake, health problems (heart disease, hypertension, high cholesterol, stroke, diabetes), quality of life of the five domains from the EuroQol 5 Dimension 3 Level (EQ-5D-3L) (mobility, self-care, usual activities, pain/discomfort, anxiety/depression), stressors (own physical health problem or condition, own emotional or mental health problem or condition), and intention to make a health behaviour change in the next year (to begin/increase PA, to increase fruit/vegetable intake, to reduce alcohol consumption, to quit smoking/reduce amount smoked, to reduce stress). Participants also self-rated their ability to handle personal crises, and handle day-to-day stress on a 5-point Likert scale (where 1 = “Poor” and 5 = “Excellent”).

#### Knowledge

The HABiT survey [22] included 19 knowledge questions in total, 11 regarding CVD and 8 regarding DM risk factors and symptoms. Each question was a statement, and the participant needed to indicate if they believed it was true or false using a 5-point Likert Scale (Definitely True, Maybe True, Not Sure or Don’t Know, Maybe False, Definitely False). If participants answered “Definitely True” or “Maybe True” when the correct answer was “True,” they were coded as correct. All other answers (“Not sure or Don’t Know,” “Maybe False,” “Definitely False”) were coded as incorrect. Participants’ answers were coded as correct for “Definitely False” or “Maybe False” when the correct answer was “False,” with all other answers coded as incorrect.

#### Self-efficacy

The self-efficacy questions in the HABiT survey [22] were adapted from the chronic disease self-efficacy scales of the Stanford Chronic Disease Self-Management Study [26]. Self-efficacy was measured by asking participants five questions about their confidence in ability to change health behaviours: “How confident are you that in the next year, you will be able to: Improve your physical activity, Increase your fruit and vegetable intake, Reduce your alcohol intake, Quit smoking, Reduce stress?” Responses were provided on a 7-point scale, where 1 corresponded to “Not at all confident” and 7 to “Extremely confident.”

### Analysis

Descriptive statistics were used to analyze the sociodemographic characteristics and risk factors for participants. Descriptive statistics were also used to analyze participant knowledge of CVD and DM risk factors and symptoms, as well as participants’ self-efficacy to make behaviour change. Subgroup analyses by risk factors such as health behaviour and chronic disease were also completed. Multivariable logistic regression models were also created for all five confidence questions. Each outcome was dichotomized with a response of 4 to 7 indicating high self-efficacy and a response of 1-3 indicating low self-efficacy. For “confidence in ability to reduce alcohol intake” and “confidence in ability to quit smoking,” participants were restricted to drinkers and smokers only, respectively. All five models included sociodemographic factors (age, gender), health risk factors, knowledge score, quality of life indicators, rating of ability to handle personal crises, rating of ability to handle day-to-day stress, and intention to complete the behaviour as independent variables. As this was an exploratory study, the primary focus of the results and discussion sections was on statistically significant findings; however, all results are informative and have been provided for the reader. Significance was determined using a *p*-value threshold of 0.05 and 95% confidence intervals have been presented. All analyses were conducted using IBM SPSS Statistics 20.0.

## Results

A total of 599 participants were included in this study (mean age = 72.24, SD: 8.61, min = 55, max = 99). The majority of participants were female (75.6%), white (89.4%), and had completed high school or less (68.7%). With regard to lifestyle risk factors, 140 participants (24.6%) were identified as drinkers, 183 (32.3%) as smokers, 273 (46.1%) as having low PA levels (less than 30 minutes a day), and 198 (33.3%) as having low fruit/vegetable intake (not every day). Among smokers, the mean number of cigarettes smoked in an average day was 17.45 (SD: 11.70). Prevalence rates of chronic disease and events associated with chronic disease among this population were 28.5% with heart disease, 54.4% with hypertension, 37.9% with high cholesterol, 12.9% having had a stroke, and 27.5% with diabetes. Participants’ mean rating of their own ability to handle personal crises on a 5-point Likert scale was 3.13 (SD: 1.21), and mean rating of their own ability to handle day-to-day stress on the same scale was 3.43 (SD: 1.08). Tables 1, 2 and 3 contain a full description of participant demographics, risk factors, and other factors.

### Health knowledge

Health knowledge was found to vary between participant subgroups. Notably, compared to the full study group, the subgroup with diabetes had a higher frequency of correct responses for statements “people who have family members with diabetes have an increased risk of developing diabetes” (88.5% versus 78.8%), “diabetes is a risk factor for heart attacks and strokes” (85.5% versus 75.1%), and “diabetes becomes more common as people get older” (77.6% versus 69.9%). For the question “diabetes can be cured,” the subgroup of current smokers and the subgroup with post-secondary education had a higher frequency of correct responses compared to the overall study population (53.6, 55.4, and 44.4% respectively). The subgroup of those who had a stroke had better knowledge than the full study population of whether “eating too much sugar and other sweet foods is a cause of diabetes,” (42.9% versus 33.1% correct) and “diabetes can cause other serious health problems” (36.4% versus 27.0%). For cardiovascular disease, those in the subgroup who had a stroke had a higher frequency of correct answers compared to the overall study population for whether “the recommended blood pressure for most adults is less than 120/80” (68.8% versus 60.3%). The subgroup with diabetes had a greater proportion of correct answers than the overall study population for “the following blood pressure is considered to be high: 140/90” (64.8% versus 55.4%). Those with high cholesterol and those with heart disease answered correctly at a higher frequency compared to the overall study group for “you can tell if you have high blood pressure (without a blood pressure cuff) because you will probably feel unwell” (57.7, 57.9, and 49.2% respectively). Tables 4, 5, 6 and 7 contain a full description of DM and CVD knowledge.

**Table 4:** Knowledge of type 2 diabetes mellitus (by demographic and lifestyle risk factor)

Outcomes		Population						
Knowledge question	Response	All respondents *n* = 599	With any post- secondary education *n* = 186	High BMI (Overweight and Obese) *n* = 388	Current drinkers *n* = 140	Current smokers *n* = 183	Not physically active (less than 30 mins daily) *n* = 273	Low fruit and vegetable intake *n* = 198
To reduce the risk of diabetes you need to eat well and exercise regularly	Correct (%)	563 (94.0)	176 (94.6)	365 (94.1)	127 (90.7)	172 (94.0)	257 (94.1)	183 (92.4)
You are at risk of developing diabetes if you are obese	Correct (%)	515 (86.0)	167 (89.8)	350 (90.2)	119 (85.0)	150 (82.0)	225 (82.4)	162 (81.8)
People who have family members with diabetes have an increased risk of developing diabetes	Correct (%)	472 (78.8)	150 (80.6)	310 (79.9)	105 (75.0)	144 (78.7)	210 (76.9)	158 (79.8)
Diabetes is a risk factor for heart attacks and strokes	Correct (%)	450 (75.1)	144 (77.4)	311 (80.2)	106 (75.7)	138 (75.4)	210 (76.9)	147 (74.2)
Diabetes becomes more common as people get older	Correct (%)	417 (69.6)	126 (67.7)	279 (71.9)	94 (67.1)	115 (62.8)	186 (68.1)	127 (64.1)
Diabetes can be cured	Correct (%)	266 (44.4)	103 (55.4)	174 (44.8)	66 (47.1)	98 (53.6)	128 (46.9)	80 (40.4)
Eating too much sugar and other sweet foods is a cause of diabetes	Correct (%)	198 (33.1)	65 (34.9)	129 (33.2)	43 (30.7)	55 (30.1)	89 (32.6)	70 (35.4)
Diabetes can cause other serious health problems	Correct (%)	62 (27.0)	57 (30.6)	114 (29.4)	37 (26.4)	49 (26.8)	78 (28.6)	54 (27.3)
Total Diabetes Knowledge Score (out of total possible 8 points)	Mean (SD)	5.08 (1.48)	5.31 (1.42)	5.24 (1.43)	4.98 (1.49)	5.03 (1.43)	5.07 (1.48)	4.95 (1.61)
Total Knowledge Score (out of total possible 19 points)	Mean (SD)	13.67 (2.80)	14.06 (2.64)	13.99 (2.60)	13.39 (2.99)	13.50 (2.71)	13.75 (2.81)	13.29 (2.84)

Less than 2% of responses were missing for each question

**Table 5:** Knowledge of type 2 diabetes mellitus (by chronic disease)

Outcomes		Population					
Knowledge question	Response	All respondents *n* = 599	With Heart Disease *n* = 171	With Hypertension *n* = 326	With High Cholesterol *n* = 227	Had a Stroke *n* = 77	With Diabetes *n* = 165
To reduce the risk of diabetes you need to eat well and exercise regularly	Correct (%)	563 (94.0)	157 (91.8)	309 (94.8)	216 (95.2)	71 (92.2)	157 (95.2)
You are at risk of developing diabetes if you are obese	Correct (%)	515 (86.0)	147 (86.0)	292 (89.6)	199 (87.7)	68 (88.3)	148 (89.7)
People who have family members with diabetes have an increased risk of developing diabetes	Correct (%)	472 (78.8)	132 (77.2)	264 (81.0)	181 (79.7)	63 (81.8)	146 (88.5)
Diabetes is a risk factor for heart attacks and strokes	Correct (%)	450 (75.1)	132 (77.2)	252 (77.3)	177 (78.0)	63 (81.8)	141 (85.5)
Diabetes becomes more common as people get older	Correct (%)	417 (69.6)	120 (70.2)	234 (71.8)	161 (70.9)	58 (75.3)	128 (77.6)
Diabetes can be cured	Correct (%)	266 (44.4)	72 (42.1)	149 (45.7)	109 (48.0)	39 (50.6)	67 (40.6)
Eating too much sugar and other sweet foods is a cause of diabetes	Correct (%)	198 (33.1)	54 (31.6)	100 (30.7)	70 (30.8)	33 (42.9)	65 (39.4)
Diabetes can cause other serious health problems	Correct (%)	62 (27.0)	49 (28.7)	83 (25.5)	56 (24.7)	28 (36.4)	47 (28.5)
Total DM Score (out of total possible 8 points)	Mean (SD)	5.08 (1.48)	5.05 (1.49)	5.16 (1.48)	5.15 (1.42)	5.49 (1.47)	5.44 (1.21)
Total Knowledge Score (out of total possible 19 points)	Mean (SD)	13.67 (2.80)	13.88 (2.70)	14.08 (2.58)	14.09 (2.44)	14.33 (2.45)	14.23 (2.54)

Less than 2% of responses were missing for each question

**Table 6:** Knowledge of cardiovascular disease (by demographic and lifestyle risk factor)

Outcomes		Population						
Knowledge question	Response	All respondents *n* = 599	With any post- secondary education *n* = 186	High BMI (Overweight and Obese) *n* = 388	Current drinkers *n* = 140	Current smokers *n* = 183	Not physically active (less than 30 mins daily) *n* = 273	Low fruit and vegetable intake *n* = 198
High blood pressure is a risk factor for heart attacks and strokes	Correct (%)	558 (93.2)	180 (96.8)	365 (94.1)	128 (91.4)	171 (94.5)	257 (94.1)	180 (90.9)
In the general population, the following things can contribute to people having high blood pressure: (i) having a stressful lifestyle	Correct (%)	555 (92.7)	180 (96.8)	365 (94.1)	127 (90.7)	169 (92.3)	256 (93.8)	181 (91.4)
High blood pressure can cause other serious health problems	Correct (%)	545 (91.0)	178 (95.7)	355 (91.5)	124 (88.6)	165 (90.2)	250 (91.6)	183 (92.4)
In the general population, the following things can contribute to people having high blood pressure: (iii) eating too much salt	Correct (%)	534 (89.1)	167 (89.8)	354 (91.2)	119 (85.0)	161 (88.0)	247 (90.5)	178 (89.9)
High blood pressure can be treated by exercise and weight loss	Correct (%)	525 (87.6)	167 (89.8)	344 (88.7)	120 (85.7)	163 (89.1)	246 (90.1)	171 (86.4)
In the general population, the following things can contribute to people having high blood pressure: (ii) drinking too much alcohol	Correct (%)	500 (83.5)	162 (87.1)	327 (84.3)	120 (85.7)	153 (83.6)	228 (83.5)	160 (80.8)
Lifestyle changes such as stopping smoking and weight loss can decrease blood pressure	Correct (%)	495 (82.6)	159 (85.5)	331 (85.3)	113 (80.7)	152 (83.1)	228 (83.5)	154 (77.8)
High blood pressure becomes more common as people get older	Correct (%)	447 (74.6)	133 (71.5)	296 (76.3)	98 (70.0)	128 (69.9)	206 (75.5)	141 (71.2)
The recommended blood pressure for most adults is less than 120/80	Correct (%)	361 (60.3)	114 (61.3)	249 (64.2)	85 (60.7)	106 (57.9)	165 (60.4)	107 (54.0)
The following blood pressure is considered to be high: 140/90	Correct (%)	332 (55.4)	106 (57.0)	220 (56.7)	79 (56.4)	95 (51.9)	152 (55.7)	103 (52.0)
You can tell if you have high blood pressure (without a blood pressure cuff) because you will probably feel unwell	Correct (%)	295 (49.2)	83 (44.6)	190 (49.0)	65 (46.4)	86 (47.0)	138 (50.5)	93 (47.0)
Total Cardiovascular Diease Knowledge Score (out of total possible 11 points)	Mean (SD)	8.59 (1.96)	8.76 (1.77)	8.75 (1.78)	8.41 (2.12)	8.48 (1.98)	8.69 (1.95)	8.34 (1.99)
Total Knowledge Score (out of total possible 19 points)	Mean (SD)	13.67 (2.80)	14.06 (2.64)	13.99 (2.60)	13.39 (2.99)	13.50 (2.71)	13.75 (2.81)	13.29 (2.84)

Less than 2% of responses were missing for each question

**Table 7:** Knowledge of cardiovascular disease (by chronic disease)

Outcomes		Population					
Knowledge question	Response	All respondents *n* = 599	With Heart Disease *n* = 171	With Hypertension *n* = 326	With High Cholesterol *n* = 227	Had a Stroke *n* = 77	With Diabetes *n* = 165
High blood pressure is a risk factor for heart attacks and strokes	Correct (%)	558 (93.2)	160 (93.6)	308 (94.5)	214 (94.3)	73 (94.8)	152 (92.1)
In the general population, the following things can contribute to people having high blood pressure: (i) having a stressful lifestyle	Correct (%)	555 (92.7)	159 (93.0)	305 (93.6)	216 (95.2)	69 (89.6)	155 (93.9)
High blood pressure can cause other serious health problems	Correct (%)	545 (91.0)	159 (93.0)	303 (92.9)	213 (93.8)	73 (94.8)	152 (92.1)
In the general population, the following things can contribute to people having high blood pressure: (iii) eating too much salt	Correct (%)	534 (89.1)	157 (91.8)	301 (92.3)	211 (93.0)	71 (92.2)	150 (90.9)
High blood pressure can be treated by exercise and weight loss	Correct (%)	525 (87.6)	157 (91.8)	294 (90.2)	205 (90.3)	71 (92.2)	146 (88.5)
In the general population, the following things can contribute to people having high blood pressure: (ii) drinking too much alcohol	Correct (%)	500 (83.5)	140 (81.9)	274 (84.0)	191 (84.1)	64 (83.1)	132 (80.0)
Lifestyle changes such as stopping smoking and weight loss can decrease blood pressure	Correct (%)	495 (82.6)	150 (87.7)	280 (85.9)	191 (84.1)	68 (88.3)	138 (83.6)
High blood pressure becomes more common as people get older	Correct (%)	447 (74.6)	125 (73.1)	255 (78.2)	173 (76.2)	57 (74.0)	128 (77.6)
The recommended blood pressure for most adults is less than 120/80	Correct (%)	361 (60.3)	106 (62.0)	212 (65.0)	148 (65.2)	53 (68.8)	102 (61.8)
The following blood pressure is considered to be high: 140/90	Correct (%)	332 (55.4)	98 (57.3)	201 (61.7)	137 (60.4)	45 (58.4)	107 (64.8)
You can tell if you have high blood pressure (without a blood pressure cuff) because you will probably feel unwell	Correct (%)	295 (49.2)	99 (57.9)	174 (53.4)	131 (57.7)	37 (48.1)	87 (52.7)
Total Cardiovascular Disease Knowledge Score (out of total possible 11 points)	Mean (SD)	8.59 (1.96)	8.83 (1.74)	8.91 (1.72)	8.94 (1.65)	8.84 (1.67)	8.78 (1.82)
Total Knowledge Score (out of total possible 19 points)	Mean (SD)	13.67 (2.80)	13.88 (2.70)	14.08 (2.58)	14.09 (2.44)	14.33 (2.45)	14.23 (2.54)

Less than 2% of responses were missing for each question

### Self-efficacy to make health behaviour change

Tables 8, 9 and 10 contain a full description of self-efficacy for each study subgroup. Table 11 contains odds ratios based on complete logistic regression models of self-efficacy. Significant (*p* < 0.05) associations were found between self-efficacy measures and some participant characteristics. Self-efficacy to increase PA was positively associated with being currently active (Odds Ratio [OR] = 1.655, 95% Confidence Interval [CI] 1.073 to 2.551) overall knowledge score (OR = 1.150, 95% CI 1.066 to 1.240), and intention to increase PA (OR = 2.141, 95% CI 1.428 to 3.210). Self-efficacy to increase fruit/vegetable intake was negatively associated with age (OR = 0.966, 95% CI 0.939 to 0.993) as well as positively associated with overall knowledge score (OR = 1.201, 95% CI 1.111 to 1.299), and intention to increase fruit/vegetable intake (OR = 2.100, 95% CI 1.247 to 3.535). Self-efficacy to reduce alcohol intake was positively associated with age (OR = 1.083, 95% CI 1.013 to 1.158). Self-efficacy to quit smoking was positively associated with rating of ability to handle personal crises (OR = 1.509, 95% CI 1.037 to 2.194) and intention to quit smoking (OR = 2.572, 95% CI 1.177 to 5.620) as well as negatively associated with having problems performing usual activities (OR = 0.267, 95% CI 0.088 to 0.813) and average number of cigarettes smoked per day (OR = 0.949, 95% CI 0.909 to 0.991). Self-efficacy to reduce stress was positively associated with rating of ability to handle personal crises (OR = 1.357, 95% CI 1.120 to 1.645).

**Table 8:** Description of self efficacy (by participant risk factor)

Outcomes			Self-efficacy (7-point Likert scale)									
			Confidence in ability to improve physical activity		Confidence in ability to increase fruit & vegetable intake		Confidence in ability to reduce alcohol intake^a^		Confidence in ability to quit smoking^b^		Confidence in ability to reduce stress	
			Mean (SD)	n	Mean (SD)	n	Mean (SD)	n	Mean (SD)	n	Mean (SD)	n
Population	All respondents		4.68 (2.03)	595	5.07 (1.95)	599	4.19 (2.52)	137^a^	3.61 (2.34)	182^b^	4.95 (2.11)	594
	BMI	Underweight	4.12 (1.93)	17	4.88 (2.03)	17	3.67 (3.06)	3	3.44 (2.79)	9	4.53 (2.43)	17
		Normal	4.58 (2.24)	154	5.19 (1.99)	155	4.39 (2.25)	2	3.97 (2.43)	60	5.13 (1.98)	152
		Overweight	4.80 (1.93)	183	5.05 (2.00)	183	4.37 (2.55)	52	3.18 (2.23)	44	5.02 (2.11)	182
		Obese	4.88 (1.84)	203	5.18 (1.79)	205	3.92 (2.70)	38	3.63 (2.20)	56	4.84 (2.09)	205
	Low fruit/vegetable intake		4.40 (2.11)	595	4.80 (1.84)	198	3.95 (2.38)	39	3.48 (2.39)	79	4.65 (2.24)	196
	Smokers		4.60 (2.03)	182	5.01 (1.80)	183	4.02 (2.48)	50	–	–	4.70 (2.14)	181
	Drinkers		4.98 (2.05)	139	5.29 (1.82)	140	–	–	3.36 (2.33)	50	4.93 (2.20)	138
	Not physically active		5.07 (1.94)	271	5.25 (1.92)	273	4.27 (2.38)	78	3.69 (2.25)	87	4.90 (2.12)	270
	Greater than median number of cigarettes smoked daily (> 15)	Above median (>15)	–	–	–	–	–	–	3.18 (2.23)	84	–	–
		At and below median (≤15)	–	–	–	–	–	–	3.98 (2.37)	92	–	–

^a^restricted to those who indicated they are drinkers

^b^restricted to those who indicated they are smokers

**Table 9:** Description of self efficacy (by participant health concern)

Outcomes	Self-efficacy (7-point Likert scale)									
	Confidence in ability to improve physical activity		Confidence in ability to increase fruit & vegetable intake		Confidence in ability to reduce alcohol intake^a^		Confidence in ability to quit smoking^b^		Confidence in ability to reduce stress	
	Mean (SD)	n	Mean (SD)	n	Mean (SD)	n	Mean (SD)	n	Mean (SD)	n
Population										
All respondents	4.68 (2.03)	595	5.07 (1.95)	599	4.19 (2.52)	137^a^	3.61 (2.34)	182^b^	4.95 (2.11)	594
With mobility problems	4.47 (2.12)	344	4.94 (2.01)	347	4.35 (2.63)	65	3.76 (2.38)	105	4.88 (2.16)	346
With self-care problems	4.24 (2.29)	111	4.55 (2.20)	113	4.80 (2.76)	15	4.26 (2.28)	31	4.85 (2.25)	113
With problems with usual activities	4.32 (2.10)	242	4.87 (2.04)	245	4.28 (2.62)	43	3.74 (2.29)	73	4.80 (2.10)	245
With pain or discomfort issues	4.57 (2.06)	401	4.99 (1.98)	405	4.08 (2.56)	93	3.61 (2.29)	137	4.77 (2.10)	403
With anxiety or depression	4.68 (2.06)	260	4.99 (1.99)	262	4.37 (2.45)	62	3.85 (2.37)	91	4.52 (2.11)	262
With stressor: Own physical health problem or condition	4.34 (2.07)	271	4.90 (1.96)	271	4.46 (2.49)	46	3.90 (2.34)	79	4.54 (2.06)	271
With stressor: Own emotional or mental health problem or condition	4.75 (1.95)	170	5.09 (1.95)	170	4.16 (2.47)	38	3.61 (2.22)	57	4.25 (2.08)	169

^a^restricted to those who indicated they are drinkers

^b^restricted to those who indicated they are smokers

**Table 10:** Description of self efficacy (by other participant factor)

Outcomes			Self-efficacy (7-point Likert scale)									
			Confidence in ability to improve PA		Confidence in ability to increase fruit & vegetable intake		Confidence in ability to reduce alcohol intake^a^		Confidence in ability to quit smoking^b^		Confidence in ability to reduce stress	
			Mean (SD)	n	Mean (SD)	n	Mean (SD)	n	Mean (SD)	n	Mean (SD)	n
Population	All respondents		4.68 (2.03)	595	5.07 (1.95)	599	4.19 (2.52)	137^a^	3.61 (2.34)	182^b^	4.95 (2.11)	594
	Knowledge score	1st Quartile	3.93 (2.20)	152	4.16 (2.12)	152	4.02 (2.58)	42	2.80 (2.28)	46	4.69 (2.32)	150
		2nd Quartile	4.74 (1.94)	182	5.19 (1.90)	184	3.68 (2.47)	41	4.10 (2.36)	63	4.93 (2.02)	182
		3rd Quartile	4.95 (1.90)	185	5.44 (1.71)	186	4.08 (2.68)	25	3.09 (2.05)	34	5.04 (2.09)	185
		4th Quartile	5.38 (1.79)	76	5.71 (1.68)	77	5.24 (2.15)	29	4.23 (2.33)	39	5.29 (1.91)	77
	Ability to handle personal crises (Likert scale of 5) below median (< 3)	At and Above median (≥3)	4.84 (1.98)	412	5.21 (1.87)	416	4.28 (2.55)	98	3.67 (2.38)	123	5.30 (1.97)	412
		Below median (< 3)	4.35 (2.08)	181	4.78 (2.10)	181	4.05 (2.44)	38	3.50 (2.28)	58	4.19 (2.20)	180
	Ability to handle day-to-day stress (Likert scale of 5) below median (< 3)	At and Above median (≥3)	4.82 (1.99)	486	5.15 (1.89)	490	4.25 (2.59)^c^	76^c^	3.59 (2.33)	148	5.11 (2.05)	485
		Below median (< 3)	4.07 (2.11)	108	4.74 (2.19)	108	4.17 (2.44)^c^	60^c^	3.68 (2.42)	34	4.28 (2.25)	108
	With Intention to complete the behaviour		5.27 (1.62)	284	5.57 (1.47)	141	3.94 (2.14)	17	4.58 (2.05)	67	4.67 (1.90)	94

^a^restricted to those who indicated they are drinkers

^b^restricted to those who indicated they are smokers

^c^median score for ability to handle day-to-day stress for those who drink = 4.00

**Table 11:** Logistic regression model of self-efficacy

Logistic Regressions	Odds Ratio (95% CI) for self-efficacy (7-point Likert scale)				
	Confidence in ability to improve physical activity	Confidence in ability to increase fruit & vegetable intake	Confidence in ability to reduce alcohol intake^a^	Confidence in ability to quit smoking^b^	Confidence in ability to reduce stress
Predictor					
Age (in years)	0.989 (0.963, 1.017)	0.966* (0.939, 0.993)	1.083* (1.013, 1.158)	1.029 (0.969, 1.093)	1.006 (0.979, 1.034)
Male (reference: female)	0.838 (0.520, 1.349)	0.700 (0.430, 1.138)	0.858 (0.326, 2.256)	1.073 (0.432, 2.665)	0.876 (0.548, 1.403)
BMI score	1.008 (0.978, 1.039)	0.990 (0.959, 1.021)	1.017 (0.937, 1.103)	0.983 (0.923, 1.047)	0.990 (0.961, 1.020)
Has fruit/vegetable intake every day	1.291 (0.840, 1.985)	1.454 (0.937, 2.256)	0.761 (0.262, 2.210)	1.187 (0.529, 2.662)	1.415 (0.927, 2.160)
Smoker	1.009 (0.637, 1.598)	0.875 (0.548, 1.395)	1.075 (0.430, 2.690)	–	0.790 (0.507, 1.233)
Drinker	1.331 (0.810, 2.187)	1.418 (0.848, 2.372)	–	1.078 (0.446, 2.607)	0.790 (0.489, 1.274)
Physically active 30 min/day	1.655* (1.073, 2.551)	1.101 (0.705, 1.718)	1.504 (0.566, 3.994)	1.221 (0.510, 2.921)	0.732 (0.475, 1.126)
Knowledge score	1.150* (1.066, 1.240)	1.201* (1.111, 1.299)	1.010 (0.862, 1.183)	1.040 (0.900, 1.203)	1.024 (0.953, 1.101)
Has mobility problems	0.739 (0.453, 1.206)	1.078 (0.647, 1.795)	0.953 (0.325, 2.797)	1.880 (0.744, 4.749)	1.141 (0.700, 1.861)
Has self-care problems	1.401 (0.792, 2.478)	0.687 (0.386, 1.222)	0.994 (0.212, 4.663)	3.062 (0.892, 10.510)	1.053 (0.602, 1.842)
Has problems with usual activities	0.820 (0.496, 1.356)	1.022 (0.604, 1.730)	0.903 (0.279, 2.923)	0.267* (0.088, 0.813)	1.006 (0.610, 1.657)
Has pain or discomfort issues	0.858 (0.529, 1.394)	0.814 (0.491, 1.35)	1.141 (0.395, 3.296)	0.751 (0.292, 1.935)	0.684 (0.420, 1.112)
Has anxiety or depression	1.086 (0.699, 1.689)	1.013 (0.641, 1.603)	1.232 (0.454, 3.342)	1.931 (0.831, 4.489)	0.831 (0.540, 1.278)
Has stressor: Own physical health problem or condition	0.776 (0.502, 1.200)	1.064 (0.675, 1.680)	2.078 (0.781, 5.524)	1.393 (0.602, 3.225)	0.701 (0.453, 1.085)
Has stressor: Own emotional or mental health problem or condition	1.315 (0.802, 2.157)	1.015 (0.610, 1.688)	0.943 (0.270, 3.294)	0.822 (0.305, 2.213)	0.790 (0.489, 1.276)
Ability to handle personal crises rating (5-point scale)	1.150 (0.947, 1.397)	1.126 (0.922, 1.374)	1.296 (0.810, 2.073)	1.509* (1.037, 2.194)	1.357* (1.120, 1.645)
Ability to handle day-to-day stress rating (5-point scale)	1.165 (0.928, 1.461)	1.063 (0.841, 1.343)	0.978 (0.561, 1.707)	0.745 (0.457, 1.217)	1.081 (0.86, 1.357)
Has intention to complete the behaviour	2.141* (1.428, 3.210)	2.100* (1.247, 3.535)	0.594 (0.168, 2.098)	2.572* (1.177, 5.620)	1.012 (0.594, 1.724)
Average number of cigarettes smoked daily	–	–	–	0.949* (0.909, 0.991)	–

^a^restricted to those who indicated they are drinkers

^b^restricted to those who indicated they are smokers

*value is statistically significant (*p* < 0.05)

Unless otherwise indicated, the reference category is 'no

## Discussion

This study explored the chronic disease knowledge, self-efficacy to improve health behaviours, and personal factors that influence self-efficacy in older adults (ages 55+) in Ontario social housing. While subgroups of residents with chronic disease had a greater knowledge of CVD and DM risk factors and symptoms, various other characteristics were associated with confidence to make health behaviour change.

### Interpretation of findings

#### Knowledge

Those with chronic disease had higher knowledge of DM and CVD risk factors and symptoms than the overall study group. This result is expected, as those with chronic disease who receive treatment likely receive health education directly from the healthcare providers who treat them. For example, the case of where the subgroup with diabetes has a greater proportion of correct answers for DM-related questions when compared to the overall study population corroborates previous research regarding diabetes knowledge among patients diagnosed with and treated for diabetes for varied periods of time [27–30], and research on patient education in primary care among those with type 2 diabetes [31–33]. In the interest of improving self-management to prevent and manage chronic disease in older adults, there is a need to increase health literacy and health knowledge in those who have not been diagnosed yet [10, 34–37]. However, having a chronic disease and receiving care from providers does not appear to be the only source for knowledge — those with post-secondary education indicated greater knowledge than the overall study population for some risk factors/symptoms, in particular, knowing whether diabetes can be cured. This greater knowledge indicates that there may be some health knowledge regarding chronic disease that is conferred by formal education, especially via development of health literacy, an association which has been found elsewhere [38–41]. Considering the low rates of post-secondary education among older adults in social housing, as well as the fact that the three knowledge questions with the lowest frequency of correct answers concerned diabetes (“diabetes can be cured,” 44.4% correct, “eating too much sugar and other sweet foods is a cause for diabetes” 33.1% correct, and “diabetes can cause other serious health problems,” 27.0% correct), it is possible that diabetes knowledge is not as accessible to older adults in social housing outside of formal education settings.

#### Self-efficacy

In the study population, the highest confidence rating was to increase fruit/vegetable intake, and the lowest was to quit smoking. Importantly, a higher proportion of smokers was found among this population with 32.3% of the older adults in social housing surveyed (74.6% of whom were ages 65+) reporting being a “smoker,” compared to 9% of older adults in Ontario (ages 65+) reporting being a current smoker [42]. It is important to note that our study defines “smoker” as both self-reported daily and occasional smokers; the Ontario Tobacco Monitoring Report defines “current smoking” as “having smoked in the past 30 days and having smoked 100 cigarettes in one’s lifetime” [42]. Additionally, Ontario populations with low education and who rent their current dwellings (both of which are characteristic of older adults in social housing) have high rates of smoking [42]. Our study results indicate that there is not a lot of confidence to mitigate, through self-management, this health risk factor that older adults in social housing disproportionately face. Based on social cognitive theory, living in this environment where smoking is a social norm, and where it is likely they have personally experienced or witnessed others unsuccessfully attempting to quit, may explain the low self-efficacy observed; however, additional research is needed to explore this hypothesis. With regard to health knowledge’s association with self-efficacy, those in our study within the lowest quartile of knowledge also had the lowest self-efficacy to make health behaviour changes compared to the other quartiles, except for confidence to reduce alcohol intake (2nd knowledge quartile was lowest confidence). Confidence scores trend upward as knowledge quartile increases, except from the 1st to 2nd quartile for confidence to reduce alcohol, and confidence to quit smoking, which increases from 1st to 3rd to 2nd to 4th quartile. Overall, however, the self-efficacy scores trend upwards with greater knowledge.

Furthermore, overall knowledge score is significantly associated with confidence to increase PA and confidence to increase fruit/vegetable intake. As such, it is possible that increasing health knowledge through education can improve self-efficacy to increase PA and fruit/vegetable intake; however, additional interventions other than health education may be needed in this population to increase confidence to reduce alcohol intake, quit smoking, and reduce stress. For self-efficacy to reduce stress, the ability to handle personal crises was positively associated; this is an expected finding [43] and suggests that interventions promoting resilience may be effective in increasing the health of this population [43]. For smoking, there were two factors positively associated with self-efficacy to quit smoking in this population: intention to quit smoking, and ability to handle personal crises. At the same time, self-efficacy to quit smoking was negatively associated with having problems with usual activities and with average number of cigarettes smoked per day. Taken together, these findings may point to smoking behaviour as coping mechanisms for dealing with stress, and the higher levels of stress as a barrier to quitting smoking, which has been described elsewhere [44].

### Strengths and limitations

Study strengths include that it reached a population that has been historically difficult to access, as low education, limited internet access, and low literacy makes traditional surveys and data collection difficult with older adults in social housing [15, 18]. The overall sample size, including its representation of older adults across various Ontario communities is also a strength, as is the use of the HABiT tool, which is validated and a reliable method of data collection with older adults in social housing. This paper adds important knowledge regarding chronic disease self-management in this vulnerable population, which is key to improving their health outcomes and population health overall.

Some limitations of this study include sample size for some subgroups; namely, drinkers (*n* = 140). Thus, analyses restricted to those who consume alcohol (including as a risk factor group for health knowledge, and confidence in ability to reduce alcohol intake) must take into account smaller sample size. Additionally, this study was cross-sectional, meaning that we cannot determine causality or temporality of associations observed. Self-reported measures were also primarily used; thus, even though HABiT is a validated tool, there is still the potential for response bias.

### Areas for future research

The disparities in health knowledge in the study population, especially greater knowledge in sub-populations with chronic diseases, suggest that interventions to develop health knowledge before chronic disease develops will benefit this vulnerable population. Such interventions may also fill a gap in knowledge that exists due to a low proportion of older adults in social housing having attended post-secondary education. Considering the association of health knowledge scores with confidence to improve PA and increase fruit/vegetable intake, interventions that increase knowledge before chronic disease develops may not only aid in preventing chronic disease development in the first place, but also increase self-management skills through improving self-efficacy to engage in behaviour change. Furthermore, programming and/or interventions to assist older adults in social housing to manage stress (for example, providing and developing tools for handling personal crises) may help build self-confidence to reduce stress and cope with stress in other ways rather than through smoking. Previous literature indicates that culturally appropriate media, including the use of storytelling, can be effective for increasing African American older adults living in affordable housing’s knowledge of CVD, DM, and development of food selection skills, stress management skills, and self-efficacy [45]. Thus, development and evaluation of an intervention specifically geared to the demographics of older adults living in Ontario’s social housing may have significant impact. Finally, low self-efficacy has been identified as a barrier to motivation to change among older adults living in social housing [46]. As our study also found links between self-efficacy and intent to change, interventions for older adults in social housing that build their self-efficacy to make health changes can help them increase intent to change health behaviours. By taking a proactive approach to health education and knowledge transfer, and development of stress management and goal/intention-setting skills, there is potential to improve self-management skills among older adults in social housing. Improved self-management skills, in turn, can result in better health outcomes for them, as well as reduced cost to the healthcare system through reduced/more appropriate use of healthcare resources.

## Conclusion

Chronic disease self-management is a challenge for older adults, especially those living in social housing who face additional challenges and vulnerabilities linked to social determinants of health. Acquisition of health knowledge regarding chronic diseases such as diabetes and cardiovascular disease holds important implications for health outcomes among older adults living in social housing. This study posits that in addition to knowledge of risk factors as an important aspect of chronic disease prevention, health knowledge can have an impact on members of this population’s self-management skills through their confidence to make health behaviour change. Furthermore, other predictors of self-efficacy depict a clearer picture of personal factors that can affect confidence to make health behaviour changes, and the types of policies and programs that can affect self-efficacy in this vulnerable population living in a setting where there may be limited role modeling successfully enacting healthy behaviours. Implementing strategies for improving health knowledge and self-efficacy to make behaviour change among older adults in social housing can improve their health outcomes, and reduce strain on Canada’s healthcare systems as the overall population ages.

## Data Availability

The data that support the findings of this study are not publicly available due to them containing information that could compromise participant privacy. De-identified, limited data will be shared by the lead author upon request.

## Abbreviations

CVD: Cardiovascular disease
DM: Type 2 diabetes mellitus
PA: Physical activity
HABiT: Health Awareness and Behaviour Tool
HRQoL: Health-related quality of life
CANRISK: Canadian Diabetes Risk Questionnaire
BMI: Body Mass Index
EQ-5D-3L: EuroQol 5 Dimension 3 Level
